# Sex Vulnerabilities to Hypoxia-Ischemia at Birth

**DOI:** 10.1001/jamanetworkopen.2023.26542

**Published:** 2023-08-01

**Authors:** Lina F. Chalak, Jessica E. Pruszynski, Catherine Y. Spong

**Affiliations:** 1 Department of Pediatrics, University of Texas Southwestern Medical Center, Dallas; 2 Parkland Health and Hospital Systems, Dallas, Texas; 3 Department of Obstetrics & Gynecology, University of Texas Southwestern Medical Center, Dallas

## Abstract

This cross-sectional study examines the incidence of hypoxic ischemic encephalopathy in male vs female neonates.

## Introduction

There is strong evidence in large meta-analyses to suggest that male infants are at increased risk for brain injury, stroke, hypoxic ischemic encephalopathy (HIE), and death,^1,2,3^ whereas individual studies were inconclusive. The protective effects of estrogen at birth affect brain structure, hormonal, neurophysiologic, and cellular responses.^3^

Our understanding of sex vulnerabilities in hypoxia-ischemia, including the effects of hypothermia, originated from preclinical work. Pooled preclinical asphyxia studies by Wood et al^1^ in the newborn rat have demonstrated sex differences in cooling using meta-analysis, while small reports failed to show any statistically significant differences.^4^

A caveat in translational models is the absence of a clinical encephalopathy phenotype. Clinical HIE trials were not powered to address sex differences with respect to sensitivity to a hypoxic ischemic insult. A recent clinical study by Sewel et al^5^ reported no associations of sex with death or disability at 18 to 22 months in a subgroup analysis of 100 infants cooled. Similarly, head cooling secondary analysis of 91 infants showed no significant differences in responses to hypothermia between sexes.^4^

## Methods

We queried our database at Parkland Health, a single inborn large tertiary care center, to test sex susceptibility differences to a hypoxic ischemic insult in a cohort study conducted between December 1, 2005, and December 1, 2020, using the STROBE reporting guideline. Universal umbilical cord gas was analyzed, with significant fetal acidosis (SFA) defined by American College of Obstetricians and Gynecologists criteria as umbilical cord pH less than 7 and/or base deficit greater than 12. Relative risks and 95% CIs were calculated for outcomes and stratified by sex. A χ^2^ test was performed for comparisons. Institutional review board approval was obtained using University of Texas Southwestern guidelines; data were deidentified. All statistical analyses were conducted using R core software, version 4.2.2 (R Foundation for Statistical Computing). Statistical significance was set at 2-sided *P* < .05.

## Results

A total of 157 538 singleton infants were born at Parkland Health during the study period. Boys represented 79 598 (51%) of the infants and girls represented 75 650 (49%) of this cohort; 155 248 (99%) had cord gas studies performed.

A hypoxic-ischemic insult with SFA was observed in 5590 infants (3.6%) during the study period. We found significant differences (*P* < .001) in the sex of SFA infants with 3019 males (54%) and 2571 females (46%) vs 51% males and 49% females without SFA. The Table shows SFA was associated with a 100-fold increased risk of HIE requiring administration of whole-body hypothermia (relative risk, 92.69; 95% CI, 64.42-133.35), as well as risk for neonatal intensive care unit admission, seizures, need for respiratory support, sepsis, and death. Subgroup analysis summarized in the Figure did not show significant differences, including 200 infants needing hypothermia (52% males, 48% females).

**Table.** Differences Between Male and Female Infants With Fetal Acidosis

Variable	Males, No. (%)			Females, No. (%)		
	Acidemia (n = 3019)	No acidemia (n = 76 579)	Relative risk (95% CI)	Acidemia (n = 2571)	No acidemia (n = 73 079)	Relative risk (95% CI)
HIE requiring hypothermia	79 (2.6)	25 (0.03)	80 (51-125)	71 (2.8)	25 (0.03)	80 (51-127)
NICU admission	285 (9.4)	1190 (1.6)	6 (5-7)	236 (9.2)	871 (1.2)	8 (7-9)
Mechanical ventilation	99 (3.3)	333 (0.4)	8 (6-9)	71 (2.8)	214 (0.3)	10 (7-12)
Sepsis	14 (0.5)	59 (0.1)	6 (3-11)	11 (0.4)	62 (0.1)	5 (3-10)
Death	15 (0.5)	51 (0.1)	8 (4-13)	13 (0.5)	42 (0.1)	9 (5-16)

Abbreviations: HIE, hypoxic ischemic encephalopathy; NICU, neonatal intensive care unit.

**Figure.**
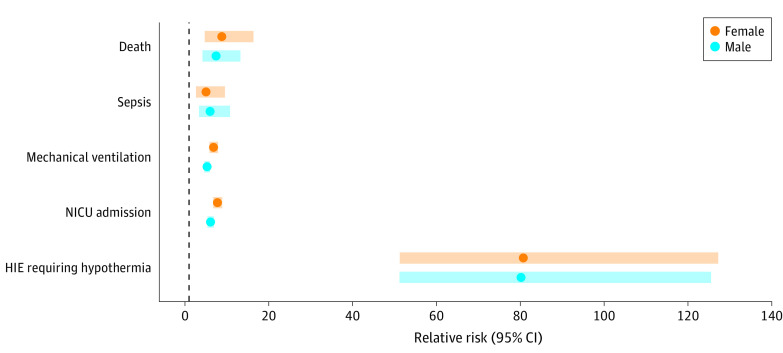
Secondary Analysis of Sex-Stratified Neonatal Outcomes HIE indicates hypoxic ischemic encephalopathy; NICU, neonatal intensive care unit.

## Discussion

This large clinical report noted increased susceptibility of males to hypoxia-ischemia, although no significant difference was detected in the subanalysis of the small group of infants requiring hypothermia, and may help bridge differences between clinical and preclinical observations. The root of the discrepancy regarding sex vulnerability in the literature is the magnitude of the difference in the incidence and severity of asphyxia and responses to therapy between males and females, although the significance is only shown with large cohorts, as in this report. This is consistent with the physiologic effects of estrogen and in large stroke studies showing sexual dimorphism in preclinical and clinical studies.

Study limitations include the lack of long-term outcomes. We cannot comment on the evolution of hypoxic-ischemic brain injury over time and response to hypothermia, which may be also modulated by sex as reported in preclinical studies.^6^ Strengths include the large size of the cohort and the use of universal cord gas screening allowing generalizability. Future protective strategies should be pooled to detect sex differences in treatment effect.

## Notes

Supplement.
Data Sharing Statement
