# Supplementary material for: Sex Vulnerabilities to Hypoxia-Ischemia at Birth
Source: JAMA Netw Open. 2023 Aug 1;6(8):e2326542. doi: 10.1001/jamanetworkopen.2023.26542 (PMC10394577; doi:10.1001/jamanetworkopen.2023.26542)
Supplement: Supplement. — Data Sharing Statement [file jamanetwopen-e2326542-s001.pdf]

Data Sharing Statement

Chalak. Sex Vulnerabilities to Hypoxia-Ischemia at Birth. *JAMA Netw Open*. Published August 01, 2023. doi:10.1001/jamanetworkopen.2023.26542

Data
Data available: Yes
Data types: Deidentified participant data
How to access data: lina.chalak@utsouthwestern.edu
When available: With publication

Supporting Documents
Document types: None

Additional Information
Who can access the data: researchers data approved
Types of analyses: research
Mechanisms of data availability: with signed agreement
Any additional restrictions: none
